# *Pantoea agglomerans* Infections in Children: Report of Two Cases

**DOI:** 10.1155/2018/4158734

**Published:** 2018-01-21

**Authors:** Shraddha Siwakoti, Rinku Sah, Rupa Singh Rajbhandari, Basudha Khanal

**Affiliations:** ^1^Department of Microbiology, B.P. Koirala Institute of Health Sciences, Dharan, Nepal; ^2^Department of Pediatrics, B.P. Koirala Institute of Health Sciences, Dharan, Nepal

## Abstract

**Introduction:**

*Pantoea agglomerans,* primarily an environmental and agricultural organism has been reported as both commensal and pathogen of humans. We present two case reports of *P. agglomerans* infections in children that involved the meninges and bloodstream.

**Case Presentations:**

A 6-month-old female baby, diagnosed as congenital hydrocephalus secondary to aqueduct stenosis with ventriculoperitoneal shunt in situ, operated 14 days back was brought to the pediatric emergency with a two-day history of high fever associated with vomiting, irritability, excessive crying, and decreased feeding. Postoperative meningitis was confirmed as cerebrospinal fluid culture revealed *P. agglomerans*. She responded well with a 14-day intravenous (IV) course of ceftriaxone. Also, we report a case of a 3-year-old male child referred to our center with a provisional diagnosis of UTI with chickenpox for further evaluation. During his 24-hour stay at the local hospital, he had received oral antibiotics and urinary catherization. Urine culture of catheter clamp urine was sterile. *P. agglomerans* was grown in blood culture. He was treated successfully with IV ceftriaxone and amikacin.

**Conclusion:**

*P. agglomerans* can cause postsurgical meningitis and bloodstream infection in children. The clinical course of infection was mild and timely administration of proper antibiotic resulted in a favorable outcome.

## 1. Introduction

*Pantoea agglomerans* is a gram-negative aerobic bacillus that belongs to the family Enterobacteriaceae. It is primarily an environmental and agricultural organism that inhabits plants, soil, and water. This bacterium has been reported as both commensal and pathogen of animals and humans [1]. Human infections may be associated with trauma caused by penetration of vegetative material and also with secondary bacteremia or nosocomial infections that are related to medical equipment such as intravenous catheters or contaminated intravenous fluids and parenteral nutrition [2–4]. Spontaneously occurring bacteremia and meningitis caused by this pathogen have rarely been reported, especially in children. Here, we present two case reports of *P. agglomerans* infections in children that involved the meninges and bloodstream.

## 2. Case Report 1: Meningitis

**Figure 1:**
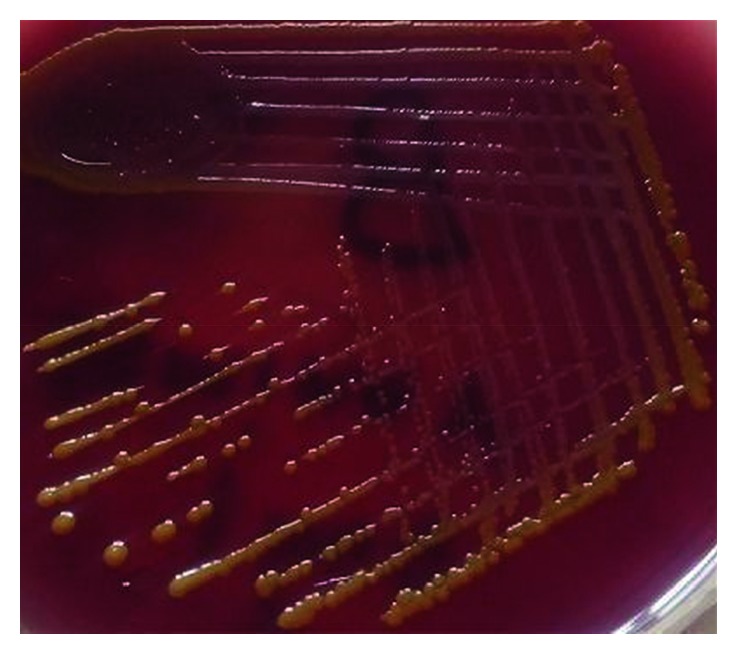
Growth of *Pantoea agglomerans* in blood agar.

This case was a six-month-old female infant, diagnosed as congenital hydrocephalus secondary to aqueduct stenosis with VP shunt in situ. Fourteen days earlier, the child had undergone ventriculoperitoneal (VP) shunt surgery. The patient presented to the pediatric emergency with a two-day history of high fever associated with vomiting (2 episodes), irritability, excessive crying, and decreased feeding. On examination, her heart rate was 136/min, respiratory rate was 60/min, and temperature was 37.7°C. Central nervous system examination showcased normal cry, hypertonia, decreased power, and exaggerated reflexes on both upper and lower limbs. Pupils were bilaterally equal and reacting to the light. Examination results of the respiratory system, cardiovascular system, and abdomen were within normal limits. The child was treated empirically with IV vancomycin and ceftriaxone after collecting the CSF sample by lumbar puncture. CSF analysis showed an elevated white blood cell count of 400 × 10^6^/L (95% polymorphs and 5% lymphocytes), raised protein level of 141 mg/dl, and decreased glucose level of 40 mg/dl (blood glucose level: 70 mg/dl). Gram stain of direct CSF smear showed few pus cells and few gram negative bacilli. CSF culture on chocolate and 5% sheep blood agar showed yellow pigmented, smooth surface colonies (Figure 1) and Mac Conkey's agar grew a nonlactose fermenting bacillus after 48-hours of aerobic incubation at 35°C. Based on biochemical reactions (Table 1), the colony was identified as *Pantoea agglomerans*. Antimicrobial susceptibility by Kirby–Bauer disc diffusion method showed the isolate to be resistant to ampicillin and sensitive to amikacin, ceftriaxone, ciprofloxacin, cotrimoxazole, and meropenem. After receiving the CSF culture report, vancomycin was discontinued, and IV ceftriaxone was continued for 2 weeks. The child's condition improved, and she was discharged after 15 days of hospital stay.

## 3. Case Report 2: Blood Stream Infection

A 3-year-old male child from the eastern part of Nepal was referred to our tertiary center after 24 hours admission at a local hospital with a provisional diagnosis of UTI with chickenpox for further evaluation. The child had a four-day history of abdominal pain, pain and difficulty with urination, urinary retention, and fever (up to 39°C). Urinary catheterization was done, and oral antibiotic was administered at the local hospital. The patient had also experienced rashes for 3 days, which started from lower limb and was later generalized. The patient had a recent history of UTI one month back. On the current admission, examination revealed temperature to be 37°C and skin showed multiple, erythematous rashes with dew drop appearance. Routine urine microscopic examination revealed the presence of 2 to 3 pus cells per high power field. Urine culture of catheter clamp urine did not yield a growth. Abdominal ultrasound showed mild hydronephrosis bilaterally and dense internal echoes within the urinary bladder lumen. A voiding cystourethrogram showed typical findings of a posterior urethral valve (PUV) with grade III vesicoureteral reflux. Blood culture grew *Pantoea agglomerans* sensitive to ampicillin, amikacin, ceftriaxone, ciprofloxacin, cotrimoxazole, and meropenem. Other laboratory parameters were within normal range. The child was empirically treated with IV ceftriaxone and amikacin which was continued for 7 days after getting the antibiotic sensitivity report. The child was successfully treated and discharged on the 8th day of hospital stay. He is currently being followed up at pediatric outpatient department and managed conservatively for PUV.

## 4. Discussion

Human infections caused by *P. agglomerans* are most often associated with wound infection with plant material or hospital acquired due to contamination of medical equipment and fluids. The most common infections caused by this pathogen in children are blood stream infection, abscess, osteomyelitis, septic arthritis, and urinary tract infection. And, the source of these infections as described in literature is due to thorn pricks, infected parenteral fluids, and indwelling catheters [4]. *Pantoea* spp. seems to be a relatively uncommon cause of meningitis. In our case, the absence of any bacteria other than *P. agglomerans* on the CSF culture confirmed that this pathogen was responsible for the postoperative meningitis. Similarly, Wang and Fraser have isolated *P. agglomerans* from the brain abscess that developed after infarction and hemicraniectomy [5]. A case report of *Pantoea calida* causing postsurgical meningitis in a 52-year-old female has been reported by Fritz et al. [6]. Our isolation of *P. agglomerans* from a similar case indicates that *Pantoea* spp. must be considered as an opportunistic Enterobacteriaceae pathogen responsible for postsurgical meningitis. This *P. agglomerans* isolate displayed *in vitro* sensitivity to all the commonly used antibiotics, and accordingly, the child recovered with proper antibiotic treatment. A similar finding of antimicrobial sensitivity pattern was reported by Fritz et al. of their *P. calida* isolate from postsurgical meningitis where the patient had full recovery after 14 days of treatment with meropenem [6]. This depicts that meningitis caused by *Pantoea* spp. can have a mild clinical course, and administration of an effective antibiotic can cure the infection.

In our second case report, *P. agglomerans* was isolated from blood culture from a child. There can be two possible causes for this blood stream infection. It is conceivable that this bacteremia could be secondary to the UTI episode. The child had clinical features of UTI and was diagnosed with PUV, an important risk factor for UTI. Microbiology testing revealed a sterile urine culture which can be explained by the recent antibiotic administration at the local hospital. Instead, this bacteremia could be an event of primary blood stream infection; by definition not secondary to localized foci, as the exact source of bacteremia could not be established. Moreover, there was no identifiable exogenous source of infection. Most of the cases of *P. agglomerans* bacteremia in the literature have been documented in association with the contamination of intravenous fluid, total parenteral nutrition, and blood products [2, 3]. Conversely, the spontaneously occurring bacteremia has rarely been reported, especially for children. A study by Cruz et al. reported 23 culture-documented *P. agglomerans* infections in children with 21 central venous line- (CVL-) related bacteremic episodes and 2 nonCVL-associated spontaneous bacteremic episodes over 6 years [4]. Likewise 5 cases of sporadic *P. agglomerans* septicemia in preterm neonates with full recovery of all cases due to proper antibiotic therapy have been reported [7]. A case report of bloodstream infection caused by *Pantoea* spp. in a vaginally delivered 4-day-old baby from India had a favorable outcome after antibiotic therapy [8]. The outcome in our case was also benign after antibiotic treatment which may be partly due to early diagnosis and the adequacy of the antibiotics. Moreover, we believe that this favorable outcome of our child was probably because of infection caused by relatively less virulent strain. In contrary, there are several previously reported cases in the literature that had a fatal outcome [3, 4, 7]. Bergman et al. reported mortality of 3 cases of sporadic septicemia out of 125 infections with *P. agglomerans* among 6,383 newborns hospitalized in an intensive care unit [9]. This lethal outcome may be due to decline of patient's immunity caused by underlying disease, prematurity, and/or hospital procedures.

To the best of our knowledge, these are the first case reports of *P. agglomerans* causing infections in children from Nepal. Being an uncommon agent of human infection, *P. agglomerans* may be underreported or reported as any other member of Enterobacteriaceae in the routine settings. Due to the ubiquitous presence of *P. agglomerans* in nature, strict compliance to infection control practices would prevent the infection with this agent.

## 5. Conclusion

*P. agglomerans* can be the opportunistic agent for postsurgical meningitis and pathogen for bloodstream infection in children. The clinical course of infection caused by this bacterium can be mild, probably due to infection caused by relatively less virulent strain and can be treated with proper antibiotic therapy.

## Figures and Tables

**Table 1:** Biochemical tests to differentiate *P. agglomerans*.

Biochemicals	Interpretation
Catalase	Produced
Oxidase	Not produced
Citrate (Simmons)	Utilized
Urease, hydrogen sulfide, indole	Not produced
Motility	Motile
Nitrate	Reduced to nitrite
Glucose, xylose, arabinose, maltose, trehalose, rhamnose, mannitol	Fermented
Lactose, sucrose, sorbitol	Not fermented
Oxidative fermentative (OF)	Fermentative
Methyl red, Voges–Proskauer	Positive
Lysine, ornithine, arginine	Not decarboxylated
